# The Aqueous Extract of* Gynura divaricata *(L.) DC. Improves Glucose and Lipid Metabolism and Ameliorates Type 2 Diabetes Mellitus

**DOI:** 10.1155/2018/8686297

**Published:** 2018-01-23

**Authors:** Jinnan Li, Jinlei Feng, Hong Wei, Qunying Liu, Ting Yang, Shengpan Hou, Ying Zhao, Bo Zhang, Cheng Yang

**Affiliations:** ^1^High-Throughput Molecular Drug Discovery Center, Tianjin International Joint Academy of Biomedicine, Tianjin 300457, China; ^2^School of Chinese Medicine, Beijing University of Chinese Medicine, Beijing 100029, China; ^3^State Key Laboratory of Medicinal Chemical Biology and College of Pharmacy, Nankai University, Tianjin 300350, China; ^4^Tianjin Key Laboratory of Molecular Drug Research, Tianjin International Joint Academy of Biomedicine, Tianjin 300457, China

## Abstract

Type 2 diabetes mellitus (T2DM) is a chronic disease characterized by hyperglycemia and dyslipidemia caused by impaired insulin secretion and resistance of the peripheral tissues. A major pathogenesis of T2DM is obesity-associated insulin resistance.* Gynura divaricata *(L.) DC. (GD) is a natural plant and has been reported to have numerous health-promoting effects on both animals and humans. In this study, we aimed to elucidate the regulatory mechanism of GD improving glucose and lipid metabolism in an obesity animal model induced by high-fat and high-sugar diet in combination with low dose of streptozocin and an insulin-resistant HepG2 cell model induced by dexamethasone. The study showed that the water extract of GD (GD extract A) could significantly reduce fasting serum glucose, reverse dyslipidemia and pancreatic damage, and regulate the body weight of mice. We also found that GD extract A had low toxicity* in vivo* and* in vitro*. Furthermore, GD extract A may increase glucose consumption in insulin-resistant HepG2 cells, markedly inhibit NF-*κ*B activation, and decrease the impairment in signaling molecules of insulin pathway, such as IRS-1, AKT, and GLUT1. Overall, the results indicate that GD extract A is a promising candidate for the prevention and treatment of T2DM.

## 1. Introduction

Type 2 diabetes mellitus (T2DM), also known as non-insulin-dependent diabetes mellitus, is a chronic metabolic disease and accounts for 90–95% of all diabetes patients [1]. The prevalence of type 2 diabetes is increasing worldwide, and it is estimated that more than 592 million people will be affected by 2035 [2]. Type 2 diabetes has the characteristics of chronic hyperglycemia and dyslipidemia caused by insulin resistance of the peripheral tissues and impaired insulin secretion from the pancreas, which is difficult to cure [3, 4]. Obesity is closely associated with an increased risk of developing insulin resistance and may be proven to be the biggest risk factor in causing T2DM [5]. Previous studies suggested that chronic inflammation may underlie the metabolic disorders of obesity-induced insulin resistance and T2DM [6, 7]. Abnormal activation in the inflammation pathway often leads to elevated expression of a variety of inflammatory factors that switch on insulin resistance phenotype, which is essential for T2DM progression [7]. For example, inhibitor of kappa B (I*κ*B) and nuclear factor *κ*B (NF-*κ*B) are often shown to be activated in the pathogenesis of inflammation-induced insulin resistance and T2DM [8, 9]. Proinflammatory stimuli induce the phosphorylation of I*κ*B, causing polyubiquitination and degradation of I*κ*B, which releases NF-*κ*B. The free NF-*κ*B translocates to the nucleus and stimulates inflammatory mediators (TNF-*α*, IL-1B, IL-6, and PKC) relevant to abnormal lipid metabolism [6, 10, 11]. TNF-*α*, IL-6, and PKC, as well as IK kinase, cause serine phosphorylation of downstream IR substrate family members IRS-1, inhibiting phosphatidylinositol 3-kinase (PI3K) and serine/threonine kinase B (AKT), which leads to insulin resistance [12]. Therefore, inhibiting the NF-*κ*B and improving IRS/p-AKT signal pathway are a promising therapeutic strategy for effective treatment of T2DM.

At present, thiazolidinediones (TZDs) are the principal class of drugs available for improvement of insulin sensitivity and inflammation in obese diabetic subjects [13]. Despite clear benefits in glycemic control, this class of drugs has recently fallen into disuse due to several severe side effects, including congestive heart failure, fluid retention, and body weight gain [14, 15]. Therefore, additional work is needed to decrease the burden of T2DM and insulin resistance. In order to search for novel drugs, many traditional herbal medicines for diabetes should be given attention. The activities of numerous herbal plants have been evaluated in animal models [1]. Previous studies have shown that some Chinese herbal medicines can be served in the search for new antidiabetic agents [16]. For example, silibinin, as the main active component extracted from milk thistle, has an antiobesity effect and protects against insulin resistance in rats of T2DM [17]. Polydatin, as the major active component of Polygonum cuspidatum Sieb. et Zucc., improves glucose and lipid metabolism in experimental diabetes through activating the AKT signaling pathway [18]. As a traditional Chinese medicine herb,* Gynura divaricata *(L.) DC. (GD) is considered as an effective medicine for the treatment of insulin resistance of T2DM. For a long time, this herb as a folk medicine has been used for the treatment of diabetes and hypertension besides other diseases [19]. Several potential therapeutic mechanisms of GD, such as the key enzymes (angiotensin-1-converting enzyme, *α*-amylase, and *α*-glycosidase) relevant to type 2 diabetes and hypertension inhibitory activity* in vitro* [20, 21] as well as hypoglycemic effect in mice [22], have been identified. A previous study demonstrated that GD improved glucose metabolism in experimental diabetic mice and rats [23, 24]. However, the detailed molecular mechanisms involved have not yet been elucidated. Thus, whether GD regulates glucose and lipid metabolism by inhibiting the NF-*κ*B and improving IRS/p-AKT pathway in experimental T2DM models has attracted our attention.

Based on known data, an insulin resistance HepG2 cell model induced by dexamethasone (Dex) and a mice model by high-fat diet (HFD) plus low dose of Streptozotocin (STZ)* in vivo* and* in vitro* were established. In this study, we systematically illustrated that GD extract A played a regulatory role in lipid and glucose homeostasis and inflammation. In addition, GD extract A improved insulin sensitivity without the common side effects of TZDs, such as body weight gain. In this present study, we investigated the detailed molecular basis underlying the antidiabetic effect of GD extract A by focusing on the transcription factor NF-*κ*B and insulin pathway. Thus, this herbal drug may not only offer better efficacy and a less side effect but also ameliorate insulin resistance. GD extract A may be a kind of potential drug for the treatment of diabetes, which has health-promoting effects.

## 2. Materials and Methods

### 2.1. Extract Preparation

Fresh mature leaves of GD (1 kg) were harvested and sampled from Bengbu city, Anhui province, China, and extracted twice with 8 L water at 100°C for 1.5 h in reflux condenser [25]. After filtering, 90% of the total combined filtrate was concentrated under reduced pressure (50°C) by a rotary evaporator (Ya Rong, Shanghai) and lyophilized to yield 26.3 g dried extract. The remaining 10% filtrate was concentrated under reduced pressure (45°C) after adding 3 L ethanol to yield 1.6 g dried extract. The precipitation yielded 1.3 g lyophilized extract. Fresh leaves of GD (0.913 kg) were extracted twice with 8 L 95% ethanol at 100°C for 1.5 h with reflux condenser [26]. After filtering, the combined filtrates were concentrated under reduced pressure (50°C) by a rotary evaporator and lyophilized to yield 17.1 g dried extract. Here, GD extracts A, B, C, and D denote the water extract, the soluble supernatant of the water extract in 75% ethanol solution, the insoluble precipitant of the water extract in 75% ethanol solution, and the ethanol extract of GD.

### 2.2. Cell Culture

Human hepatoma HepG2 cells [25, 27, 28] (ATCC, USA) were maintained in high glucose (25 mM) Dulbecco's modified Eagle's medium (DMEM) supplemented with 10% (v/v) fetal bovine serum (FBS) (ExCell, China) and 1% penicillin/streptomycin and cultured at 37°C under 5% CO_2_. After serum-starving, cells were stimulated with or without 0.5 *μ*M Dex for 24 h and then incubated with different doses of the extracts of GD. The cells were collected after different measurements of insulin (Sigma, USA) stimulation for 24 h.

### 2.3. Glucose Consumption

HepG2 cells were seeded in 96-well plates at a concentration of 1 × 10^4^ cells per well and treated with serum-free high glucose (25 mM) DMEM overnight. Cells were pretreated with or without 0.5 *μ*M Dex for 24 h and then washed three times with phosphate-buffered saline and stimulated with 1 nM insulin for 24 hours. GD extract A and pioglitazone were used to treat the insulin-resistant cells to assess the effects of GD extract A and pioglitazone on glucose uptake. After incubation with varying concentrations of GD extract A and pioglitazone, culture supernatant was sampled for glucose concentration measurement by glucose oxidation method (Jiancheng, Nanjing, China), and then glucose consumption was calculated by subtracting the glucose concentration in blank group (no cells, only medium) and normalized by MTT assay.

### 2.4. MTT Assay

The 3-(4,5-dimethylthiazol-2-yl)-2,5-diphenyltetrazolium bromide (MTT, Sigma, USA) assay was used to detect cell viability of NIH-3T3/HEK-293 cells after the medicine. Briefly, cells were seeded in 96-well plate and incubated with GD extract A for 24 h. After medium was removed, 100 *μ*l MTT (0.1 mg/ml) was added to each well and the plates were incubated for additional 4 h. MTT was then carefully removed, avoiding disturbing the mazan crystals formation. Dimethyl sulfoxide (DMSO, 150 *μ*l, Sigma, USA) was added and the absorbance of solubilized blue for mazan was read at a wavelength of 492 nm by a microplate reader (BioTek, USA) [28].

### 2.5. Animal Experiments

44-Week-old healthy specific pathogen-free male Kunming (KM) mice were obtained and were kept in the departmental animal house under controlled conditions of temperature of 20°C ± 5°C, relative humidity of 40%–70%, and a light and dark cycle of 12 h each [29]. All animal experiments were carried out according to the Standards for Laboratory Animals (GB14925-2001), and all animal procedures were approved by the Animal Ethics Committee of Tianjin International Joint Academy of Biomedicine with the following reference number: 20141204-01.

Animals were fed a standard diet (55% carbohydrate, 24% protein, 5% fat, 3% fiber, 0.6% calcium, 0.3% phosphorus, 6.1% moisture, and 6% ash w/w) and high-fat diet (20% sugar, 10% lard oil, 1% sodium cholate, 2.5% cholesterol, and 66% normal commercial pellet diet). The standard and high-fat diets were purchased from Beijing Vital River Laboratory Animal Technology Co., Ltd. After acclimatization, all animals except normal controls were allowed to have access to the high-sugar/high-fat diet. After 10 days of HFD administration, overnight-fasted (12 h) mice were given a single injection of freshly prepared STZ (60 mg/kg, i.p., in 0.1 M sodium citrate buffer (prepared on the ice and protected from light), pH 4.5, Sigma, USA) [30]. The normal control mice were given the same volume of 0.1 M sodium citrate buffer. Blood samples were collected from the tail vein 72 h after STZ treatment, which assessed for hyperglycemia by measuring fasting serum glucose level. The mice with a fasting serum glucose level above 11.1 mM were considered as diabetics in the study [31].

After establishment of diabetes model, mice were randomly divided into four groups (I–IV), and each group contained 10 animals. The following treatment schedule started and the treatments were administered for 40 days: Group I: normal control mice given saline (5.0 ml/kg, p.o.) once a day; Group II: diabetic mice given saline (5.0 ml/kg, p.o.) once a day, which served as diabetic controls; Group III: diabetic mice given the GD extract A (100 mg/kg, p.o.) once a day; Group IV: diabetic mice given pioglitazone (30 mg/kg, p.o.) once a day and this group of mice was called TZD mice. During the gavage period, mice were continually allowed free access to water and standard laboratory chow for normal control mice or high-fat chow for high-fat-diet-treated mice.

### 2.6. Glucose Tolerance Test (GTT)

A glucose tolerance test (GTT) was employed to evaluate the ability to respond appropriately to glucose challenge. After overnight fast, all mice were fed with glucose (2 g/kg, b.w.). Glucose levels were measured at 0, 30, 60, and 120 min after glucose administration. Area under the curve (AUC) was calculated using trapezoidal rule.

### 2.7. Biochemical Analysis

The mice were weighed every day. Before the end of the experiment, all mice were fasted for 12 h and terminated after anesthesia. Blood samples of 5 ml were collected and serum was separated and stored at −80°C until use. Triglyceride (TG), total cholesterol (TC), low-density lipoprotein cholesterol (LDL-C), and high-density lipoprotein cholesterol (HDL-C) in serum were detected by commercial available kits supplied by Nanjing Jiancheng Bioengineering Institute. Serum insulin levels and glucagon-like peptide-1 (GLP-1) were measured by corresponding ELISA kits (Nanjing, China).

### 2.8. Histological Assessments

Soon after termination, liver, kidney, and pancreas of each mouse were isolated. The tissues were then processed and embedded in paraffin for thin section by microtome. These sectioned slices of these tissues were then stained with hematoxylin and eosin for histological analysis (NAS scoring) under a light microscope (Nikon, Japan).

### 2.9. Luciferase Reporter Assays

For NF-*κ*B assay, HEK-293T cells were plated in 96-well plates with DMEM (10% FBS). After incubation for 6 h, cells were cotransfected with NF-*κ*B-luc and PRL-TK reporter plasmids using Lipofectamine 2000 (Invitrogen). 24 h after transfection, wells were randomly divided into three groups (*n* = 3): Group I: control group; Group II: TNF-*α* group (cells were incubated with 10 ng/ml TNF-*α* for 24 h); Group III: TNF-*α* + GD group (cells were incubated with 10 ng/ml TNF-*α* and 10 mg/ml GD extract A for 24 h). Firefly and* Renilla* luciferase activities were measured by a Dual-Luciferase Reporter Assay Kit (Promega) according to the instructions. Firefly luciferase activity was normalized to* Renilla* luciferase activity, and thus the relative luciferase activities were shown.

### 2.10. Western Blotting

Cells treated with corresponding agents were harvested and lysed in lysis buffer (50 mM Tris-HCl, 150 mM NaCl, 2 mM EDTA, 2 mM EGTA, 25 mM NaF, 25 mM b-glycerophosphate, pH 8.0, 0.2% Triton X-100, 1 mM PMSF, 10 mg/ml leupeptin, and 10 mg/ml aprotinin), as previously described. Lysate samples with equal amounts of protein were subjected to 12% (v/v) sodium dodecyl sulfate-polyacrylamide gel electrophoresis (SDS-PAGE) and subsequently transferred to polyvinylidene difluoride (PVDF) membranes. After blocking with 5% nonfat dry milk in TBST at room temperature for 1 h, membranes were incubated with corresponding rabbit polyclonal antibodies against IRS-1, p-AKT (Ser473), and GLUT1 (Affinity, Canada.) followed by appropriate horseradish peroxidase-conjugated secondary antibodies. The protein bands were visualized using a Western blotting detection system according to the manufacturer's recommendations.

### 2.11. Statistical Analyses

All data were presented as Mean ± SD and statistically analyzed by GraphPad Prism 5.0 software. Statistical comparisons between groups were made by unpaired Student's *t*-test. Independent experiments were performed at least in triplicate. A value of *P* < 0.05 or *P* < 0.01 was considered statistically significant.

## 3. Results

### 3.1. GD Extract A Ameliorates Glucose Metabolism in Dex-Induced Insulin-Resistant HepG2 Cells

To observe the effect of GD on glucose metabolism in Dex-induced insulin-resistant HepG2 cells, the glucose oxidase method was used to measure medium glucose and its conversion for glucose consumption.

**Figure 1:**
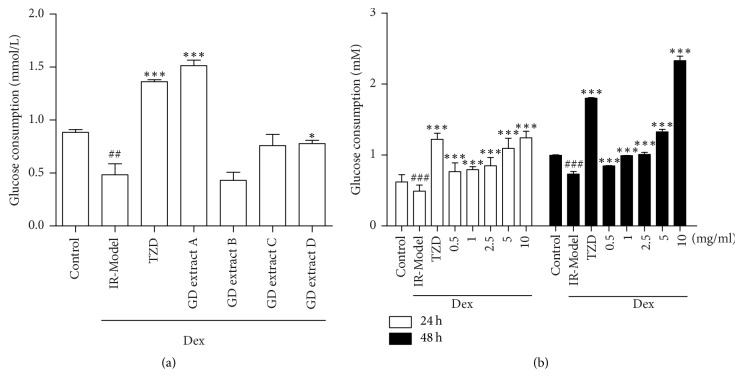
The effect of GD on glucose metabolism in Dex-induced insulin-resistant HepG2 cells. HepG2 cells were incubated with or without 500 nM Dex for 24 hours and in 1 nM insulin with or without the drugs (pioglitazone or different extracts of GD) for another 24 hours (a). HepG2 cells were incubated with or without 500 nM Dex for 24 hours and in 1 nM insulin with or without the drugs (pioglitazone or different concentrations of extract A) for another 24 hours (b). ^#^*P* < 0.05, ^##^*P* < 0.01, and ^###^*P* < 0.001 versus control group; ^*∗*^*P* < 0.05 and ^*∗∗∗*^*P* < 0.001 versus IR-Model group.

All the four plant extracts were tested at the concentration of 10 mg/ml to assess their impact on basal and Dex-stimulated glucose uptake into differentiated HepG2 cells. After incubation for 24 hours, extract B (Figure 1(a)) failed to enhance Dex-stimulated glucose uptake. However, it was observed that extract A enhanced glucose uptake significantly, as compared to the control model (Figure 1(a)). Thus, extract A ameliorated glucose metabolism significantly in insulin-resistant HepG2 cells and was evaluated for further research.

Figure 1 shows that glucose uptake and glucose consumption were significantly decreased in Dex-induced insulin-resistant HepG2 cells. The 5 and 10 mg/ml of GD extract A treatment significantly increased glucose uptake (Figure 1(b)). These results indicate that GD extract A attenuates the insulin resistance of HepG2 cells by increasing glucose uptake into the cells and promoting glucose consumption.

### 3.2. GD Extract A Reduces the Body Weight of Diabetic Mice

**Figure 2:**
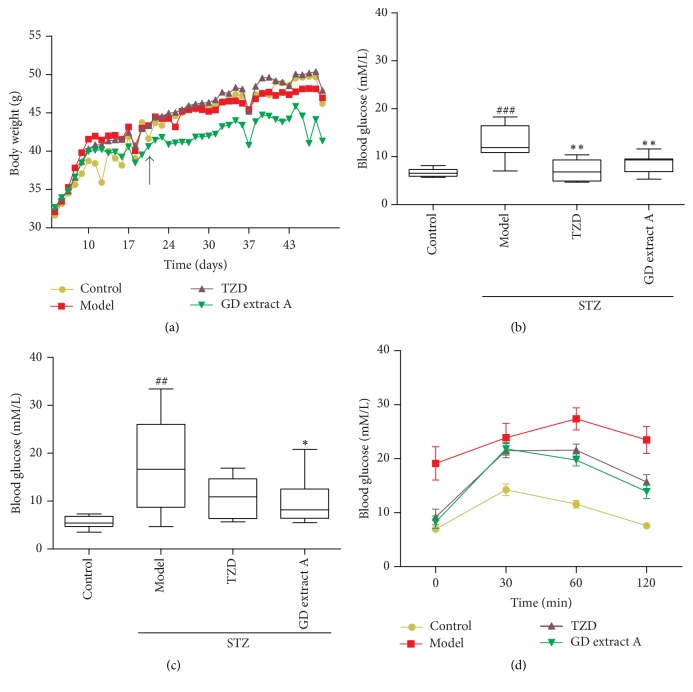
The effects of GD extract A on mice. The body weight was recorded every day (a). The blood glucose of mice after treatment with GD extract A at 100 mg/kg/day, pioglitazone at 0.03 g/kg/day, or saline (control mice) for 2 consecutive weeks (b) or 4 consecutive weeks (c). The data of GTT was collected at 0, 30, 60, and 120 min after the injection of glucose (d). ^##^*P* < 0.01 and ^###^*P* < 0.001 versus control group; ^*∗*^*P* < 0.05 and ^*∗∗*^*P* < 0.01 versus Model group. The arrow refers to the time point of the drug.

After being fed with high-fat diet for 6 continuous weeks, the mean body weight of TZD mice was higher than that of control mice. After treatment with GD extract A for 4 consecutive weeks, the body weight of the GD extract A groups was significantly reduced compared to that of diabetic mice (Figure 2(a)).

### 3.3. GD Extract A Reduces Fasting Blood Glucose Levels in Mice

STZ is used to destroy pancreatic *β*-cells and thereby induces insulin-deficient diabetes in animal models. After treatment with high-fat diet and STZ, the mean level of fasting glucose was significantly higher in diabetic mice compared to the control mice (data not shown). After treatment with GD extract A or pioglitazone for two weeks, the levels of fasting glucose index in TZD group and GD extract A group were significantly reduced compared to the diabetic mice (Figure 2(b)). After treatment with GD extract A or pioglitazone for four weeks, the fasting glucose level was all significantly reduced compared to the diabetic mice (Figure 2(c)).

### 3.4. GD Extract A Can Regulate Glucose Tolerance of Experimental Diabetic Mice

Insulin resistance and glucose metabolism dysfunction are typical clinical symptoms of metabolic syndrome. Therefore, the possible beneficial effects of GD extract A were evaluated. Diabetic mice, after being injected with D-glucose, showed higher peak blood glucose level and slower correction process than control mice. Although not completely, cotreatment with GD extract A drastically ameliorated the glucose metabolism impairment induced by insulin resistance (Figure 2(d)). Area under the curve (AUC) of control is 21.663 h·mmol/L, AUC of model is 44.374 h·mmol/L, AUC of pioglitazone is 35.544 h·mmol/L, and AUC of GD extract A is 34.66 h·mmol/L.

### 3.5. GD Extract A Can Regulate Lipid Levels of Experimental Diabetic Mice

**Figure 3:**
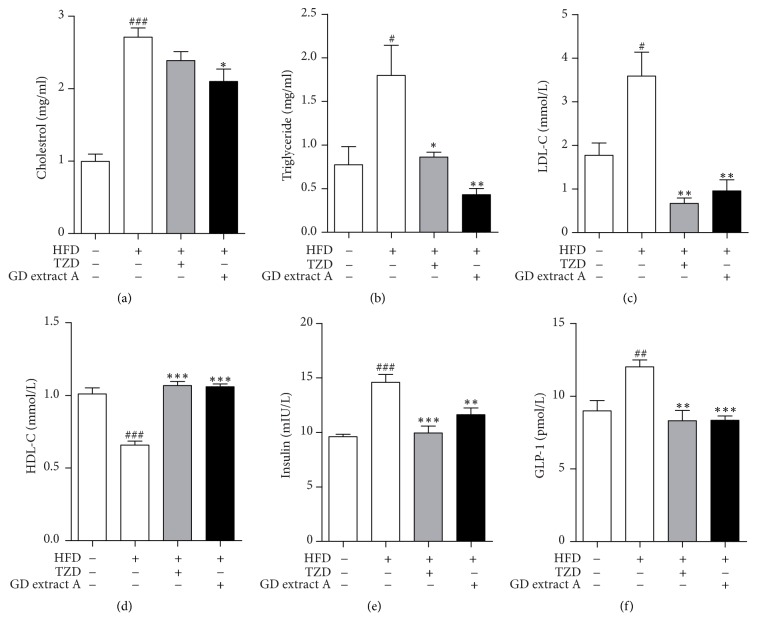
Blood biochemical indexes (cholesterol, triglyceride, LDL-C, HDL-C, insulin, and GLP-1) of diabetic mice. Blood samples were collected from the orbital sinus, and cholesterol (a), triglyceride (b), LDL-C (c), HDL-C (d), insulin (e), and GLP-1 (f) levels were measured following administration of GD extract A at 100 mg/kg/day, pioglitazone at 0.03 g/kg/day, or saline (control and model mice) for 4 consecutive weeks in high-fat-diet-treated mice. ^#^*P* < 0.05, ^##^*P* < 0.01, and ^###^*P* < 0.001 versua control group; ^*∗*^*P* < 0.05, ^*∗∗*^*P* < 0.01, and ^*∗∗∗*^*P* < 0.001 versus Model group.

As illustrated, the TC, TG, and LDL-C levels were significantly increased and HDL-C level was significantly reduced in the diabetic model compared with those of the normal control. The treatment of GD extract A and pioglitazone markedly reduced TC, TG, and LDL-C levels (Figures 3(a), 3(b), and 3(c)) and increased HDL-C level (Figure 3(d)) compared with the diabetic model group.

### 3.6. GD Extract A Reduces the Levels of Insulin and GLP-1 in Diabetic Mice

Compared to the control mice, the levels of fasting insulin and GLP-1 were significantly higher in diabetic mice (Figures 3(e) and 3(f)). After treatment with high dosage of GD extract A or pioglitazone, the levels of insulin and GLP-1 were all significantly reduced (Figures 3(e) and 3(f)).

### 3.7. GD Extract A Improves Liver, Kidney, and Pancreas Histology

**Figure 4:**
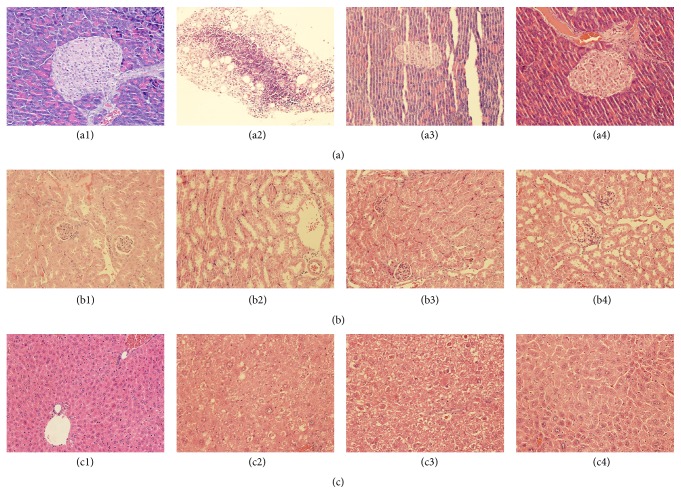
Histopathological changes in the experimental groups. Light microscopic study of pancreatic islets (a1)–(a4), kidney (b1)–(b4), and liver (c1)–(c4) from different experimental groups: control: (a1), (b1), and (c1); model: (a2), (b2), and (c2); pioglitazone: (a3), (b3), and (c3); GD extract A: (a4), (b4), and (c4).

H&E staining of liver, kidney, and pancreas section was performed. As shown in Figure 4, the liver and pancreatic islets were characterized by a large number of fat depositions in diabetic mice. The treatment with GD extract A and pioglitazone reduced the numbers of both fat droplets. GD extract A treatment potently alleviated pancreas injury.

### 3.8. GD Extract A Reduces the NF-*κ*B Protein Activation in 293T Cells

**Figure 5:**
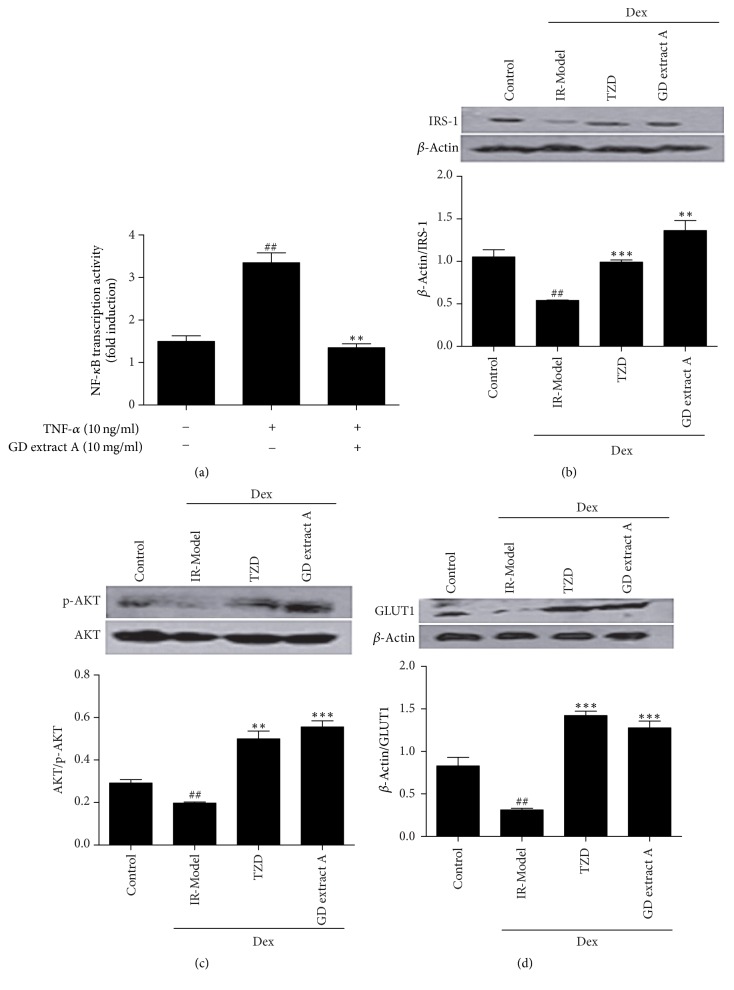
Effect of GD extract A and pioglitazone on insulin signaling pathways. NF-*κ*B transcription activity with or without TNF-*α* (10 ng/ml) and GD extract A (10 mg/ml) in 293T cells was detected by luciferase reporter assays (a). HepG2 cells were incubated with or without 100 nM Dex for 24 hours and in 1 nM insulin with or without the drugs (pioglitazone or GD extract A) for another 24 hours. Cells lysates were separated by SDS-PAGE and subjected to Western blot analysis with IRS-1 (b), anti-phosphorylation AKT antibody (Ser473) (c), and GLUT1 antibody (d). ^##^*P* < 0.01 versus control group; ^*∗∗*^*P* < 0.01 and ^*∗∗∗*^*P* < 0.001 versus IR-Model group.

Previous studies have documented that reduced I*κ*B and increased NF-*κ*B binding activity were strongly correlated with reduced insulin sensitivity [6, 8]. In order to explore the mechanism of GD extract A, luciferase reporter assays were used to measure NF-*κ*B protein activation in 293T cells. As shown in Figure 5(a), NF-*κ*B protein activation with TNF-*α* was significantly increased compared to control cells. After treatment with GD extract A, NF-*κ*B protein activation was significantly reduced compared to 293T cells with TNF-*α*.

### 3.9. Improvement of T2DM by GD Extract A Is Partially Attributed to the Modulation of IRS-1/PI3K/AKT Pathway

Previous studies have documented that the signaling pathway of IRS-1/PI3K/AKT directly transduced extracellular insulin signals to downstream pathological pathways that led to the occurrence of T2DM [32]. AKT, as a vital mediator of insulin-induced glucose and lipid metabolism, initiates glucose transporter (GLUT) translocation and intracellular glucose metabolism [33]. To explore the effect of GD extract A on activating IRS-1/PI3K/AKT pathway and improving insulin resistance, Western blot analysis was used to measure the levels of IRS-1, p-AKT, and GLUT1 in HepG2 cells. As shown in Figures 5(b)–5(d), the expressions of IRS-1, p-AKT, and GLUT1 protein were significantly reduced compared to control cells. After treatment with GD extract A and pioglitazone, the expressions of IRS-1, p-AKT, and GLUT1 protein were significantly increased compared to IR cells.

### 3.10. GD Extract A Has Low Cytotoxicity on HEK-293 and NIH-3T3 Cells

**Figure 6:**
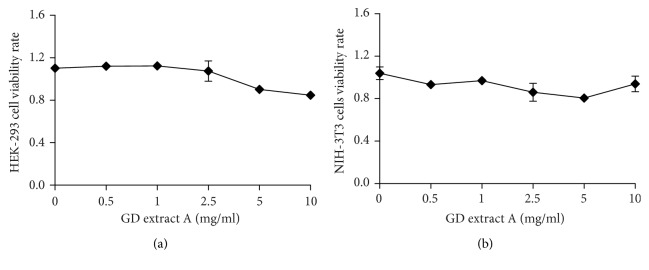
Cytotoxicity of GD extract A. Cytotoxicity of GD extract A on (a) HEK-293 and (b) NIH-3T3 cells. Standard error bars represent three independent experiments, and each experiment was performed in triplicate.

The cytotoxic effects of GD extract A on HEK-293 and NIH-3T3 cells were assessed via a cytotoxicity assay. GD extract A showed negligible toxic effect on cell viability at its highest concentration (Figure 6). This result confirmed that GD extract A had low cytotoxicity.

## 4. Discussion and Conclusions

To date, although many drugs for T2DM have been tested in clinical trials, few of them received satisfactory results in terms of therapeutic efficacy maximization and adverse effect minimization [34]. Recently, emerging evidence suggests the possible application of herbal derivatives in the treatment of T2DM, in both preclinical studies and clinical trials, such as green tea [35], Berberine [36], resveratrol [37], and coffee [38]. Several previous studies found that GD treatment indicated potential antihypertension and renoprotective effects of the herb [39]. This study revealed that the Chinese herbal formula GD could attenuate T2DM by improving the content of glucose and lipids, as well as the insulin resistance.

In this study, a model of HepG2 cells was developed using Dex and used to assess the potential of GD extract A in insulin resistance reversal. TZDs, as insulin-sensitizing drugs, reverse Dex-induced insulin resistance by suppressing the adverse effects of Dex on insulin sensitivity and glucose tolerance. This study determined that GD extract A could also reverse the Dex-induced impairment in glucose uptake. Similar to pioglitazone, GD extract A showed potential reversal of Dex-induced insulin resistance as evidenced by the restoration of glucose uptake. GD extract A and pioglitazone showed similar effect on insulin resistance. However, to our surprise, GD extract B and extract C have no glucose consumption effect. We suppose three reasons: (1) there are more hypoglycemic ingredients in GD extract A than extracts B and C; (2) glucose consumption effect may appear when the amount of these hypoglycemic ingredients is enough; (3) these hypoglycemic ingredients in GD extract A have synergistic effect on glucose consumption.

The chronic consumption of high-fat diet can induce insulin resistance in humans and animals [40–42], and it is closely related to the occurrence and development of obesity [43]. The high-fat diet and low-dose STZ induced type 2 diabetic mice model, which gives rise to a mild *β*-cell dysfunction and impairment of insulin secretion and even results in hyperglycemia [44]. In the present work, the mice in type 2 diabetes model showed significant increase in serum levels of glucose, TC, TG, and LDL-c and decrease in HDL-c, coupled with impaired glucose tolerance and insulin sensitivity when compared with the normal control group. These results indicated the successful development of type 2 diabetes mice model with insulin resistance.

Administration of GD extract A induced time-dependent changes in biochemical parameters in type 2 diabetic mice. Treatment for 4 weeks with GD extract A was found to significantly decrease the high serum glucose concentration, TC, TG, and LDL-c and increase HDL-c compared with the model group, resembling the effect of pioglitazone. GTT also verified that GD extract A markedly improved glucose tolerance and insulin resistance. And a trend towards a decrease in serum insulin concentration and GLP-1 could be found in the GD extract A group compared with the model group. This indicates that GD extract A has an effect on glucose and lipid metabolism, which does not rely on the increased insulin secretion and GLP-1 secretion. At the same time, we identified an increase in body weight in the group of pioglitazone, and this was not found in groups administrated with GD extract A.

Some studies pointed out that chronic inflammation was an important cause of obesity-induced insulin resistance [45]. Tumor necrosis factor-*α* (TNF-*α*), a cytokine associated with cachexia in cancer, is elevated in obese adipose tissue in rodents and the inhibition of this cytokine improves glucose tolerance and insulin sensitivity [46]. The adipose tissue macrophages (ATMs) can span the spectrum range from an M1 proinflammatory state that contributes to insulin resistance to an M2-polarized state in lean animals which may protect adipocytes from inflammation [3]. In obesity, these cells secrete a number of different cytokines, such as TNF-*α* and interleukin-1*β* (IL-1*β*). In turn, these cytokines can act through paracrine mechanisms to directly inhibit insulin action in insulin target cells and phosphorylation I*κ*B, which activate NF-*κ*B and then phosphorylate IRS-1 on inhibitory serine residues to result in impaired downstream signaling and increased very-low-density lipoprotein (VLDL) synthesis [47, 48]. Therefore, we used TNF-*α* to activate NF-*κ*B protein overexpression. After treatment with GD extract A, NF-*κ*B protein activation was significantly reduced compared to 293T cells with TNF-*α* only by luciferase reporter assays. In the present study, we supposed that GD extract A could improve both insulin resistance and lipid metabolism by reducing NF-*κ*B protein expression, which regulated downstream PI3K/AKT signal transduction.

**Figure 7:**
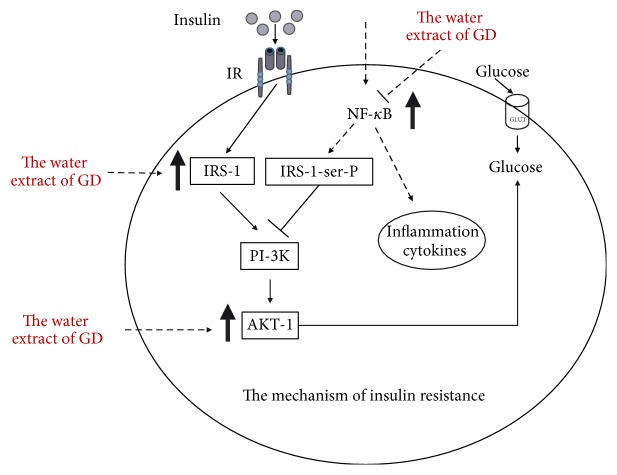
Schematic summary of mechanism of action of GD extract A.

Defects in the insulin signaling cascade, which lead to impaired glucose utilization, are believed to play a key role in the pathogenesis of insulin resistance [49]. It is conceivable that IRS-1 tyrosine phosphorylation in response to insulin stimulation generally increased the association of IRS-1, resulting in increased PI3-kinase activity, which led to activation of AKT and GLUT and ultimately to an enhancement in insulin-stimulated glucose disposal [50]. The results of this research revealed that the insulin receptor was impaired, producing an insulin-resistant state in HepG2 cells with Dex. The expression of the IRS-1 protein and IRS-1-associated p-AKT activity in HepG2 cells were significantly decreased. After treatment with GD extract A, the expressions of IRS-1 protein, p-AKT, and GLUT1 were increased. Here, the results revealed that the GD-mediated recovery of insulin action was related to the improvement of the IRS-1 signaling pathway in insulin-resistant HepG2 cells. It appeared that GD extract A may improve insulin signaling pathway and induce the subsequent increase in insulin sensitivity. The results showed that GD extract A effectively restored Dex-induced desensitization by restoring IRS-1, AKT phosphorylation, and GLUT1 activity (Figure 7).

In conclusion, GD extract A can inhibit NF-*κ*B activation. GD extract A can also improve glucose and lipid metabolism and reverse the insulin resistance without significantly increasing body weight. Therefore, the study provides a basis for future exploration of the therapeutic potentials of GD extract A in prevention and treatment of type 2 diabetes mellitus.
